# Trapped by the Lack of Control Over Savings: Evidence From Pakistan

**DOI:** 10.3389/fpsyg.2022.867841

**Published:** 2022-05-18

**Authors:** Elisa van Dongen, Syedah Ahmad, Robert Lensink, Annika Mueller

**Affiliations:** Department of Economics, Econometrics and Finance, Faculty of Economics and Business, University of Groningen, Groningen, Netherlands

**Keywords:** financial inclusion of women, saving, women’s control over resources, women’s empowerment, Pakistan

## Abstract

Gender discrimination and associated social norms are important contributing factors to the high frequency of women trapped in poverty – particularly in developing countries. Financial inclusion, especially access to formal saving services, has recently received much attention from the development community for its potential to lift women out of poverty and reduce inequality. To date, however, the impacts of social norms on women’s ability to use and benefit from such formal saving services are not widely understood. The purpose of this paper is to advance the understanding of this relationship, by investigating, in a setting where social norms put women at a disadvantage, the association between their decision-making power with respect to a newly opened formal savings account at a bank and the amount of savings kept in that account. We use data on 1,798 married women in Pakistan, from an intervention to encourage savings account uptake among them. Focusing on the usage, 8 months after the intervention, of 512 newly opened bank accounts, we find that women with at least joint control over the bank account save statistically significantly more in this account than women without any control. On average, this difference amounts to 2,339 PKR (22.40 USD), which is substantial considering that the majority of women in our sample are from lower-middle income class households and are rarely the household’s main income earners. This finding has important implications for future research, as well as for policy makers and practitioners providing financial services to women in gender unequal contexts.

## Introduction

Around the world, women are more likely to be afflicted by poverty than men (United Nations, 2020, 2021). Gender gaps in literacy and educational attainment, labor force participation, wages, and – importantly – access to finance are key contributing factors and particularly pronounced in developing countries (Bernhardt et al., 2018; Munoz Boudet et al., 2018; OECD Development Centre, 2019; World Economic Forum, 2019). Sizable gender gaps also exist in household consumption,^1^ due to women’s inferior intra-household bargaining power over financial (and other) resources (Grown, 2014; Lambert et al., 2014; Munoz Boudet et al., 2018, Women’s World Banking [WWB], 2018).

Thus, improving the financial inclusion of women, defined as increasing account ownership among women (Demirgüç-Kunt et al., 2018; World Economic Forum, 2021), has attained a prominent place on the international development agenda (Ashraf et al., 2006; Bruhn and Love, 2009; World Bank, 2012; Dupas and Robinson, 2013a; UN Women, 2016; UNCDF, 2021), as account ownership is expected to increase women’s ability to access and control financial assets,^2^ thus strengthening their intra-household bargaining power.

However, interventions targeted at increasing (savings) account ownership among women often do not consider how gender norms that correlate with positions of low bargaining power might affect usage patterns and thus, the effectiveness of these accounts in enhancing women empowerment.^3^ Indeed, even the basic premise that women who have opened such an account will then use it, is increasingly called into question: Multiple studies have documented considerable take-up, but low usage rates (see, e.g., Dupas and Robinson, 2013a,b; Kast and Pomeranz, 2014; Prina, 2015; Allen et al., 2016; Brune et al., 2016; Dupas et al., 2016; Schaner, 2016). Thus, in order to advance women empowerment through financial inclusion (savings accounts in particular) a more in-depth understanding is needed of how – in the first place – gender norms/different levels of bargaining power impact usage patterns of these accounts; and in turn, their ability to strengthen women’s bargaining power and ultimately, contribute to lifting them out of poverty.

In this study, we provide evidence regarding the extent to which the use of a newly opened savings account depends on the level of women’s bargaining power over that account. More specifically, we analyze female clients’ perception of the degree of control they exercise over a newly opened micro-savings account and its association with the amount saved in this account. Our study uses data from Ahmad et al. (2020) on 1,798 married, relatively lower income women in Punjab, Pakistan. This data was collected between September 2017 and July 2018. The participants were randomly assigned to various interventions designed to encourage them to open a bank account with Meezan Bank, a commercial Islamic bank in Pakistan. Our hypothesis is that women who have at least some degree of control over their newly opened formal savings account will accrue higher amounts of savings in the account, as, e.g., money saved in the account will be less susceptive to the demands of others, and the women will likely derive increased utility from these savings in this case.

We find that women who have joint or full control over their newly opened savings account saved an additional 2,339 PKR (22.40 USD or approximately 11 percent) on average in the first 8 months after the interventions compared to women who have no control. This amount is equivalent to roughly half an average month’s income for a person in the middle-income class in Pakistan.^4^ The observed difference in savings is thus considerable, taking into account that (i) a sizable fraction of households in our sample earns a *combined* household income below the 2015 upper-middle income line of 5,624 PKR per adult per month (World Bank, 2020),^5^ and (ii) fewer than 4 percent of the women in our sample are the main income earners of their respective households.

Our study contributes to two broad strands of academic literature on women’s empowerment and financial inclusion. Women’s empowerment is a complex concept with multiple levels (e.g., personal, household, community, and societal) and dimensions (e.g., psychological, political, legal, and economic). Hence, the exact meaning of the concept varies across and even within academic disciplines (Malhotra and Schuler, 2005, p. 80). Yet, an important and recurrent notion across all disciplines is women’s ability to make choices with respect to the use of resources. The discipline of psychology commonly proxies this ability with indicators for self-efficacy (Malhotra and Schuler, 2005, p. 83; Brody et al., 2017), while the primary focus in economics lies on the ability to access, own and control financial assets (Malhotra and Schuler, 2005, p. 83; Brody et al., 2017; World Economic Forum, 2021), such as formal savings.^6^ When discussing this ability, economists strongly emphasize the importance of relational empowerment, proxied by intra-household bargaining power (Malhotra and Schuler, 2005, p. 83; Brody et al., 2017; Huis et al., 2017). For instance, in developing countries, women generally have substantially less control over assets, including financial assets (Deere and León, 2003; Doss et al., 2015; Kieran et al., 2015; Ambler et al., 2017). An increase in assets (e.g., savings) owned and controlled by a woman strengthens her bargaining power and enables her to adjust household allocations toward her preferences (Manser and Brown, 1979; McElroy and Horney, 1981; Ambler et al., 2017).

We are not the first who draw attention to the relationship between women’s empowerment and control over resources in the form of savings. Anderson and Baland (2002) emphasize that wives often participate in informal rotating saving clubs (ROSCAs), thereby protecting their savings against claims by their husbands for immediate consumption and gaining decision-making power over their money. A related strand of literature discusses control over finances in the context of observability, e.g., Ashraf (2009) finds that under asymmetric information, women and men alike attempt to hide their money from their partner/spend immediately on consumption (i.e., increase control over these financial resources), whereas under symmetric information they are more inclined to transfer the money to the partner with most financial control. Other work that recognizes the importance of control over savings in the context of women’s empowerment analyzes the effects of varying product characteristics, e.g., commitment features, ATM access, and withdrawal fees. Schaner (2016), for instance, focuses on the influence of transaction costs on account use. She finds that for women with low bargaining power, the use of savings accounts is enhanced when withdrawal is only possible at the bank branch – and not possible *via* ATMs – thereby placing a barrier on others accessing those savings. Access to private and secure formal saving services may also in general enable women to decrease pressures from household members to share earnings or to spend it right away (Anderson and Baland, 2002; Schaner, 2016; Buvinic and Jaluka, 2018), enhancing their decision-making power and economic independence.

Our research adds to this literature, by studying a setting where the features of the account impart a high degree of control to the women (e.g., the account being registered solely in their own name, ATM access being prohibitively costly,^7^ and a photo-ID being needed for making transactions at the bank branch). Yet, our results show that even in such a case, a substantial share of married female clients indicates having no control over their account, and that married women without control save significantly and substantially less in their account. Hence, our findings imply that what seem to be the most straightforward responses to the findings in the literature, e.g., providing single-owned accounts, insufficiently address the issue of bank account usage, in particular for those women who are least empowered to begin with. The study therefore highlights that solutions to the issue of low actual savings of women with low bargaining power must be more holistic than these product design features alone.

Besides, this paper’s contribution to the literature on women empowerment, the study adds to the literature on the effectiveness of financial inclusion in reducing poverty. Now that empirical evidence has documented a lower impact of including the poor in micro credit programs on welfare opportunities (Banerjee et al., 2015), development efforts are shifting toward improving access to micro saving services (Roodman, 2012; Karlan et al., 2014; UNCDF, 2021). Savings are generally able to reduce poverty among women and gender inequality through enhancing women’s ability to manage their financial risks, smoothen consumption, and invest in their future productivity and income (Christen and Mas, 2009; Demirgüç-Kunt et al., 2017). Note that women seem to prefer to rely on savings over investments, borrowed capital and insurance, as such saving services involve less risk (Croson and Gneezy, 2009; Buvinic and Jaluka, 2018). Namely, voluntary savings do not oblige clients to make mandatory deposits and instead of requiring interest payments or fees – which is the case with microcredit programs – clients could earn interest or Mudarabah (Vonderlack and Schreiner, 2002). In short, this study’s particular focus on bargaining power with respect to saving services is highly relevant for financial inclusion in the context of women’s empowerment.

## Background, Data, and Methods

### Context, Partner Organizations, and Savings Account

The location of our study – Multan district in Pakistan – is particularly suitable considering our topic. Namely, the gender gaps in Pakistan – including the gender gap in access to finance and legal protection – are among the largest in the world (World Economic Forum, 2019). Considering the gender gap in access to finance, 29 percent of Pakistani men but only 6 percent of Pakistani women owned an account from a financial institution in 2017 (Global Findex Database, 2017). Only 10 percent of Pakistani men and just 2 percent of Pakistani women saved with formal financial institutions (Global Findex Database, 2017).

Our partner organizations in Pakistan were ‘*Meezan Bank*,’ a commercial Islamic bank, and ‘*Akhuwat*,’ an Islamic microfinance institution. With 760 branches in 223 cities, Meezan Bank is the 6th largest bank of Pakistan (Meezan Bank, 2019). The bank offers formal financial services that are Shariah-compliant. The ‘*Asaan Savings Account*’ is specifically designed for low-income unbanked or under-banked segments of the population (Meezan Bank, 2019). The Asaan Savings Account works on the basis of a Mudarabah-based relationship. In other words, Meezan Bank partially invest the stored savings and the customer earns a pre-determined share of the returns on these investments. Further, the account’s name and description indicate that the opening process is relatively swift and does not require submitting a proof of income. To open an account, a valid Computerized National Identity Card is necessary. The applicant must further complete an account opening form and illiterate participants need to bring a colored photograph for opening a photo-account. The account opening fee is 250 PKR. This fee consists of a mandatory minimum balance of 100 PKR and a 150 PKR fee for a check book with 10 checks that can be used for withdrawals or payments. The check book is also used as a log book to track savings balances.

Akhuwat is an Islamic MFI that offers Sharia-compliant microcredit products, as well as education and health services. Akhuwat was established in 2001 and started providing Sharia-compliant (and thus also interest free) microcredit to the poor with the aim to enhance their living standards. In 2017–2018, the MFI had 791 branches spread over 435 cities throughout Pakistan. Akhuwat’s primary loan category is the Family Enterprise loan, constituting about 85–90 percent of their sales in microcredit products (Akhuwat, 2020).

### The Participants and Data Collection Procedure

The study’s sample includes applicants for Akhuwat’s Family Enterprise loan, who received approval of their applications at the start of the study. At the time of the study, Akhuwat had 25 branches in Multan district (Akhuwat, 2020). One branch was randomly selected for focus group discussions, that took place approximately 1 month prior to loan disbursement meetings in which the saving accounts were offered. Data was collected from all 2,220 successful loan applicants of the remaining 24 branches. These 2,220 applicants, who were evenly distributed over the other 24 Akhuwat branches in the Multan district, were present at the loan disbursement meetings held from September 16–22, 2017. Prior to these meetings, they had been randomly selected into eight different intervention groups (see Supplementary Material Section A2). Each intervention group received a unique combination of encouragements to open an Asaan savings account with Meezan Bank during the meeting: receipt of a subsidy (‘*Subsidy’*), assistance with completing the application form (‘Help’) and a religious speech versus a conventional speech (‘R_speech’ versus ‘C_speech’). The participants could then open a savings account with Meezan Bank any time after their loan disbursement meeting concluded. Participants who were offered a subsidy as encouragement received the amount when opening the savings account up until the 31st of October 2017. The saving accounts offered are single-owned, meaning that the accounts are registered solely in the name of the account owner (i.e., the respondent in our study). The attrition rate of 5 percent is rather low.

In total, three surveys were conducted. The baseline survey was set out from mid-September 2017 to the end of October 2017. *Via* the baseline survey data on the participants’ demographic and socio-economic traits such as age, marital status, household size, literacy, and household income were collected. The follow-up survey was conducted from mid- to end December, approximately 3 months after the baseline survey. With this survey, data was collected on the participants’ self-reported control over the account using the following survey question: *“Can you by yourself decide how to use the money that accrues on the bank account?”* The endline survey was conducted at the end of June, 2018. During the endline survey interviews, data was collected on participant’s monthly savings balances – including the months March, April, May, and June – from Meezan Bank’s administrative data and the participants’ check (log)books. In the study by Ahmad et al. (2020) further details about the provided encouragements to open an account and the data collection process can be found.

This study focuses on the self-reported control over savings of married women. Therefore, this study’s sample incorporates married female respondents (*N* = 1,801), excluding men (*N* = 283) and unmarried women (*N* = 136).^8^ Of the 1,801 married women in the sample, 523 opened the single-owned Asaan savings account, 1,275 did not and 3 dropped out. In short, the final sample of this study contains data of 1,798 married female participants.

### Sample Characteristics

Table 1 summarizes the characteristics of the 1,798 married female individuals in our sample. For instance, the average married woman in our sample is 39 years old, lives in a household of 7 people, of which 5 are adults.

**TABLE 1:** Summary statistics final sample.

Variables	*N*	Mean	St. dev.	Min	Max
Age	1,798	39.30	9.30	17	59
HH_Size	1,798	6.22	2.49	1	20
N_Sons	1,745	0.48	0.92	0	9
HH_Income	1,798	0.31	0.46	0	1
Borrower_Income	1,463	1.62	0.68	1	3
Main_Earner	1,789	0.82	0.39	0	1
Read_Urdu	1,798	0.83	0.88	0	2
Grades_Passed	1,676	3.47	3.94	0	14
First_Loan	1,798	0.65	0.48	0	1
Formal_Save	1,797	0.14	0.35	0	1
Self_Empl	1,770	0.45	0.50	0	1
Not_Work	1,770	0.19	0.39	0	1

*Control, self-reported control over the savings in the Meezan Bank account; Age, age of the respondent in years; HH_Size, number of people (excluding respondent) living in the house; N_Sons, number of participant’s sons. HH_Income, household’s combined income > 30,000 PKR, where 1 = household’s combined income > 30,000 PKR, and 0 = household’s combined income ≤ 30,000 PKR; Borrower_Income, participant’s income, 0 = participant’s income ≤ 10,000 PKR, 1 = participant’s income between 10,000 and 20,000 PKR, 2 = participant’s income > 20,000 PKR; Main_Earner, household member who earns the most income within the household, where 1 = respondent or spouse, and 0 = parents (in law) or other; Read_Urdu, ability to read, where 2 = read easily, 1 = read with difficulty, and 0 = not able to read; Grades_Passed, highest grade in school completed; First_Loan, respondent is taking first loan from Akhuwat, where 1 = ‘yes’ (i.e., the respondent has never taken out a loan from Akhuwat before) and 0 = ‘no’ (i.e., the respondent has had a loan from Akhuwat before); Formal_Save, have some formal savings before experiment, where 1 = yes and 0 = otherwise; Self_Empl, self-employed, where 1 = yes and 0 = no; Not_Work, not working, where 1 = not working and 0 = working. For fuller explanations of these variables, see Supplementary Table A3.*

More than seventy percent of the married female individuals in our sample describes their financial situation over the previous year as fair, poor, or very poor. Approximately 31 percent of the households earn more than 30,000 PKR a month (289 USD).^9^ For 20 percent of the married female individuals in our sample, the parents (in law) earn most of this household income. Considering that (i) on average households consist of about 7 people among which there are 5 adults, and (ii) the upper middle income class poverty line is 5,624 PKR per adult per month (World Bank, 2020), a substantial fraction of the households is in the lower middle-income class or poor. Half of the participants indicate to have a personal income of below 10,000 PKR a month (97 USD), 39 percent earns between 10 and 20 thousand PKR and only 10 percent earns above 20 thousand PKR. A substantial share (47 percent) of the respondents earns their income through self-employment. Most self-employment activities relate to garments and embroidery work, cosmetics, or food. Nineteen percent of the participants do not work, and the remaining 34 percent earn income through salaried employment.^10^

The largest share of participants did not receive any formal education or only very little. Only about a third of the married female individuals in our sample is able to read in Urdu easily, 20 percent are able to read in Urdu with difficulty and 48 percent is not able to read in Urdu at all. Further, approximately 45 percent of the married female individuals in our sample did not pass any grade in school. On average, the married female individuals in our sample completed only 3 out of 10 grades in school.

Further, almost all participants indicated to be Muslim (99.7 percent). Only 6 married female individuals in our sample indicated to be Christian.^11^

In terms of saving behavior, only a small share (14 percent) saved formally before the experiment started. Of the 1,798 married women in the sample 523 opened an account with Meezan Bank and 1,275 did not.

In most aspects, the married women who opened a savings account with Meezan bank do not significantly differ from the married women who did not. The three differences significant at 5 percent are with respect to age, reading ability and the possession of other formal savings prior to the experiment. On average, married women that opened an account were slightly older, had a slightly lower ability to read and were less often in possession of another formal savings account. Although the number of significant differences between account openers and non-openers is rather low, these might lead to sample-induced bias. To correct for the potential selection effects, a Heckman selection model will be used (see the section Empirical Strategy). The dependent variable of interest – total savings in the Meezan Bank account – is obtained for 513 married, female individuals in our sample who opened an account.

Of these 513 women, one refused to answer the survey question inquiring who controlled the account. Thus, data on the variable *control over the Asaan savings account* is available for 512 women. Most women have at least joint control over the accrued savings in the account: 76 percent indicate to share decision-making power and 2 percent indicate to have full decision-making power over these savings. 21 percent of these married female respondents indicate to have no decision-making power over the accrued savings at all, despite the account being registered solely in her name.

Between the group of married female respondents with at least some decision-making power and the group of married female respondents with no decision-making power, the only significant difference at the 5 percent level is in the frequency of applying for a first loan with Akhuwat (Table 2). This coefficient of first loan is rather small and since we test for quite a number control variables, the significance could be due to chance. Overall, Table 2 suggests the groups with some decision-making power and no decision-making power appear to be sufficiently similar for further analysis.

**TABLE 2:** Summary statistics by Asaan savings account and level of control.

	Opened -account	No account			Control	No control		
Variables	Mean	Mean	Difference (absolute)	*N*	Mean	Mean	Difference (absolute)	*N*
Age	40.4	38.8	1.56***	1798	40.3	40.8	0.498	512
	(9.07)	(9.36)	(0.482)		(9.05)	(9.02)	(0.983)	
HH_Size	6.31	6.17	0.129	1798	6.36	6.21	0.157	512
	(2.39)	(2.53)	(0.129)		(2.31)	(2.61)	(0.259)	
N_Sons	0.472	0.486	0.015	1745	0.474	0.476	0.002	506
	(0.892)	(0.934)	(0.049)		(0.892)	(0.910)	(0.098)	
HH_Income	0.33	0.31	0.0206	1798	0.34	0.29	0.046	512
	(0.47)	(0.46)	(0.024)		(0.473)	(0.456)	(0.051)	
Borrower_Income	1.587	1.63	0.044	1463	1.578	1.62	0.042	406
	(0.665)	(0.689)	(0.040)		(0.892)	(0.685)	(0.082)	
Main_Earner	0.805	0.820	0.015	1798	0.815	0.757	0.058	512
	(0.397)	(0.384)	(0.020)		(0.042)	(0.019)	(0.043)	
Read_Urdu	0.767	0.861	0.094**	1798	0.780	0.748	0.033	512
	(0.85)	(0.86)	(0.045)		(0.852)	(0.87)	(0.093)	
Grades_Passed	3.23	3.57	−0.334	1676	3.26	3.23	0.031	480
	(3.86)	(3.97)	(0.211)		(3.86)	(3.92)	(0.435)	
First_Loan	0.683	0.642	0.041*	1798	0.659	0.785	0.126**	512
	(0.465)	(0.479)	(0.025)		(0.475)	(0.412)	(0.050)	
Formal_Save	0.092	0.16	0.068***	1797	0.086	0.103	0.016	512
	(0.288)	(0.366)	(0.018)		(0.281)	(0.305)	(0.031)	
Self_Empl	0.432	0.453	0.021	1770	0.435	0.419	0.016	505
	(0.495)	(0.498)	(0.026)		(0.496)	(0.496)	(0.054)	
Not_Work	0.209	0.176	0.033*	1770	0.195	0.257	0.062	505
	(0.407)	(0.381)	(0.020)		(0.397)	(0.439)	(0.445)	

*Control, self-reported control over the savings in the Meezan Bank account; Age, age of the respondent in years; HH_Size, number of people (excluding respondent) living in the house; N_Sons, number of participant’s sons. HH_Income, household’s combined income > 30,000 PKR, where 1 = household’s combined income > 30,000 PKR, and 0 = household’s combined income ≤ 30,000 PKR; Borrower_Income, participant’s income, 0 = participant’s income ≤ 10,000 PKR, 1 = participant’s income between 10,000 and 20,000 PKR, 2 = participant’s income > 20,000 PKR; Main_Earner, household member who earns the most income within the household, where 1 = respondent or spouse, and 0 = parents (in law) or other; Read_Urdu, ability to read, where 2 = read easily, 1 = read with difficulty, and 0 = not able to read; Grades_Passed, highest grade in school completed; First_Loan, respondent is taking first loan from Akhuwat, where 1 = ‘yes’ (i.e., the respondent has never taken out a loan from Akhuwat before) and 0 = ‘no’ (i.e., the respondent has had a loan from Akhuwat before); Formal_Save, have some formal savings before experiment, where 1 = yes and 0 = otherwise; Self_Empl, self-employed, where 1 = yes and 0 = no; Not_Work, not working, where 1 = not working and 0 = working. For fuller explanations of these variables, see Supplementary Table A3.*

***p < 0.01, **p < 0.05, *p < 0.10.

### Empirical Strategy

The aim of our empirical strategy is to measure the association between the dependent variable of interest – ‘Total savings in the Asaan savings account’ – and married women’s degree of control over the Asaan savings account. The primary estimation specification of our study is as follows:


(1) $$S_{i}^{*}=x_{i}^{\prime }\,\beta_{1}+\varepsilon_{i}$$

In the primary specification, $S_{i}^{*}$, i.e., ‘Total savings in the Meezan Bank account,’ is defined as the closing balance in May; approximately 8 months after the loan disbursement meetings. Data on participant’s monthly savings balances was collected from Meezan Bank’s administrative data and the participants check (log)books during the endline survey. The participant needs this check (log)books for making withdrawals and payments.

Next, $x_{i}^{\prime }$ includes the independent variable of interest – *Control over the savings in the Asaan savings account* – and control variables. The variable ‘*Control’* is binary; indicating ‘0’ if the respondent has no control over the savings and indicating ‘1’ if the respondent has at least some control over the savings. 79 percent of respondents has joint or full control, and 21 percent has no control over how the savings accrued in the account are used. Some control implies that the respondent decides over the use of the accrued savings (i) on their own, (ii) together with her husband or (iii) together with her husband and other household members.^12^ As mentioned in Section “The Participants and Data Collection Procedure,” the variable ‘*Control’* is based on self-reported data, collected approximately 3 months after the loan disbursement meetings took place.

Besides, $x_{i}^{\prime }$ includes the following set of control variables: age, household income, household size and ability to read. For household income, a dummy-variable is included, which equals one if the household income is above 30,000 PKR and equals zero if below 30,000 PKR. Household size is defined as the number of people (excluding respondent) living in the house. Ability to read is captured by an ordinal variable, which is equal to ‘2’ if the participant indicates to be able to easily read in Urdu, ‘1’ if the participant indicates to be able to read in Urdu with difficulty, and ‘0’ if the participant is not able to read in Urdu.

Note, however, that the dependent variable in (Eqn. 1) is only observed for participants that decided to open an account in the first place. Hence, a standard linear regression would be based only on the self-selected, non-random sample of married females who decided to open an account. Since those who opened an account may, on average, systematically differ from those who did not open an account, the group of account openers may be unrepresentative of the population we aim to analyze. For instance, literate women may be more inclined to open an account than non-literate women. Using a simple ordinary least squares (OLS) estimation based solely on the subsample of account openers may therefore lead to coefficients that suffer from sample selection bias.

To investigate whether such sample selection problem exists and if so, correct for it, we implement a Heckman selection model, which features a selection specification in addition to (Eqn. 1). The next paragraphs explain the intuition behind this approach as well as the main features of the selection specification. A more detailed description of the technicalities can be found in Supplementary A4.

The Heckman selection model allows us to test and correct for the potential biases caused by the non-random sample selection issue described above. Specifically, in addition to estimating a primary specification (Eqn. 1), of ‘Total savings in the Asaan savings account’ on women’s control over their account, the Heckman model estimates a “selection specification” (Eqn. 2 in Supplementary A4) that explicitly models the sample selection process. Briefly, it estimates the probability of opening an account based on a number of covariates (e.g., the received encouragements) using data of both married female account holders and non-account holders, and implements a correction (i.e., penalty) in the primary estimation of amount of savings in the Asaan savings account on the women’s level of control over the account and covariates (Eqn. 1). When this correction-term is of negligible size (i.e., not statistically significant), it indicates that there is no evidence for a sampling-selection problem and the primary estimation specification can be estimated simply by OLS.

## Results

### Primary Results

**FIGURE 1:**
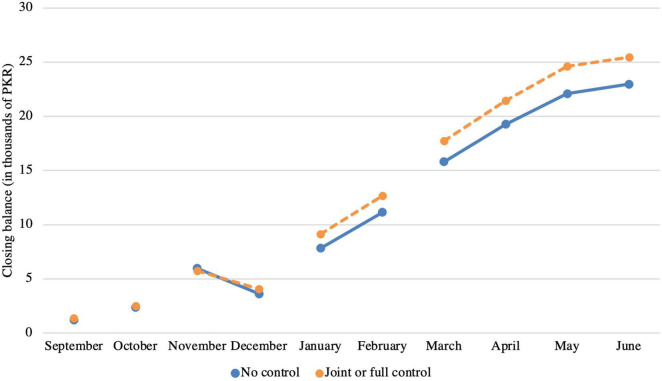
In the months September and October, the number of accounts in which at least one deposit was made equaled 67 and 254 repectively. In November and December, this number equaled 280. In January and February, this number equaled 396. From March onwards, the number of accounts equaled 512.

First, simple comparisons of the closing balances from March to June are made between the married female respondents who have at least some control and the married female respondents who do not have any control. Figure 1 displays the trends in closing balances of these two groups over the duration of the study. The mean difference between the respondents’ first deposit amount is negligibly small (32 PKR). Respondents with joint control deposit on average 1,256 PKR and respondents without control deposit on average 1,224 PKR the first time. From the figure it can be seen that over time respondents who have at least some control accrue more savings in the account on average than respondents without any control. In May 2018, the difference in total savings (i.e., the closing balance) between respondents with control and respondents without any control has grown considerably, amounting to 2,513 PKR (24 USD). Hence, respondents with control save on average approximately 11 percent more than respondents without. Considering that the purchasing power conversion factor for private consumption in Pakistan was approximately 34 LCU per international dollar (World Bank Database, 2021), these 24 USD are a considerable difference for the married women in our sample.

Next, the Heckman selection model is estimated to test for a positive association between ‘*Control*’ and ‘*Total savings in the Asaan savings account*.’ The null-hypothesis states that there is no association between the two variables. The alternative states that there is an association between the two variables. Table 3 shows the results of the Heckman selection model estimations.

**TABLE 3:** Heckman selection model estimations.

Variables	(1) Heckman selection model		(2) Heckman clustered SE		(3) Heckit model	
***S* (total savings)**						
Control	2,339	(*p* = 0.056)	2,339	(*p* = 0.094)	2,337	(*p* = 0.056)
	(1222)		(1397)		(1222)	
Age	32.2	(*p* = 0.562)	23.3	(*p* = 0.656)	31.96	(*p* = 0.565)
	(55.51)		(72.20)		(55.50)	
HH_Income	3,541	(*p* = 0.001)	3,541	(*p* = 0.007)	3,540	(*p* = 0.001)
	(1075)		(1320)		(1075)	
Read_Urdu	643.7	(*p* = 0.278)	643.7	(*p* = 0.265)	646.6	(*p* = 0. 276)
	(593.8)		(577.6)		(593.4)	
HH_Size	−105.7	(*p* = 0.615)	−105.7	(*p* = 0.560)	−105.7	(*p* = 0.615)
	(−210.0)		(181.5)		(210.0)	
Formal_Save	−3,254	(*p* = 0.066)	−3,254.1	(*p* = 0.128)	−3,238	(*p* = 0.067)
	(1772)		(2136)		(1769)	
Constant	18,934	(*p* = 0.000)	18,934	(*p* = 0.000)	18,994	(*p* = 0.000)
	(3124)		(4325)		(3097)	
**Savings account**						
R_speech_Subsidy_Help	1.87	(*p* = 0.000)	1.872	(*p* = 0.000)	1.88	(*p* = 0.000)
	(0.145)		(0.206)		(0.145)	
R_speech_Subsidy	1.11	(*p* = 0.000)	1.11	(*p* = 0.000)	1.11	(*p* = 0.000)
	(0.142)		(0.199)		(0.143)	
R_speech_Help	0.66	(*p* = 0.000)	0.67	(*p* = 0.000)	0.66	(*p* = 0.000)
	(0.146)		(0.175)		(0.146)	
R_speech	0.43	(*p* = 0.005)	0.43	(*p* = 0.081)	0.42	(*p* = 0.005)
	(0.148)		(0.244)		(0.148)	
C_speech_Subsidy_Help	0.63	(*p* = 0.000)	0.630	(*p* = 0.000)	0.636	(*p* = 0.000)
	(0.148)		(0.082)		(0.148)	
C_speech_Subsidy	0.28	(*p* = 0.078)	0.278	(*p* = 0.003)	0.289	(*p* = 0.067)
	(0.158)		(0.095)		(0.158)	
C_speech_Help	0.35	(*p* = 0.025)	0.347	(*p* = 0.024)	0.361	(*p* = 0.019)
	(0.155)		(0.154)		(0.154)	
Age	0.011	(*p* = 0.004)	0.011	(*p* = 0.018)	0.011	(*p* = 0.004)
	(0.004)		(0.004)		(0.004)	
HH_Income	0.025	(*p* = 0.624)	0.025	(*p* = 0.597)	0.025	(*p* = 0.621)
	(0.073)		(0.073)		(0.074)	
Read_Urdu	−0.020	(*p* = 0.735)	−0.019	(*p* = 0.732)	−0.020	(*p* = 0.739)
	(0.040)		(0.037)		(0.040)	
HH_Size	0.012	(*p* = 0.388)	0.012	(*p* = 0.455)	0.012	(*p* = 0.379)
	(0.014)		(0.015)		(0.014)	
Formal_Save	−0.24	(*p* = 0.027)	−0.236	(*p* = 0.123)	−0.244	(*p* = 0.025)
	(0.107)		(0.153)		(0.106)	
Constant	−1.75	(*p* = 0.000)	−1.75	(*p* = 0.000)	−1.76	(*p* = 0.000)
	(0.206)		(0.233)		(0.206)	
**Rho**	0.115	(*p* = 0.285)	0.115	(*p* = 0.382)		
	(0.109)		(0.131)			
**Lambda**	1,300		1,300		1,249	(*p* = 0.291)
	(1234)		(1474)		(1184)	
**Observations**	1,786		1,786		1,786	

*This table reports the output of the two applications of the Heckman-selection model. Column 1 represents the estimation with the Heckman selection model. Column 2 represents the Heckman selection model estimation with standard errors clustered on the branch level. Column 3 represents the Heckman two-step selection model (i.e., Heckit model). R_speech, religious speech. C_speech, conventional speech. Subsidy, receipt of subsidy for opening the account. Help, assistance with filling in application forms.*

The coefficient for ‘*Control*’ is positive and statistically significant at the 10 percent level. The *p*-value of this coefficient equals 0.056, meaning that the coefficient is very close to being statistically significant at the 5 percent level as well. The output of the Heckit model and the Heckman selection model with clustered standard errors also report positive and statistically significant coefficients for ‘*Control*’ at the 10 percent level. Based on these outcomes, one may conclude that married female individuals who have at least some decision-making power over how the savings in the Asaan savings account are used have statistically significantly higher closing balances in their Asaan savings account. The 2,339 PKR difference in average savings balance is equivalent to roughly half an average month’s income for a person in the middle-income class in Pakistan. As previously mentioned, this difference is substantial, considering that (i) a sizable fraction of households in our sample is poor or in the lower-middle income class, and (ii) fewer than 4 percent of the women in our sample are the main income earners in their respective households.

Further, the output suggests that the selection bias due to the truncation of the dependent variable is negligible. The *p*-value of the likelihood ratio test of independent equations is 0.285. Hence, we fail to reject the null hypothesis of no selection bias (ρ = 0). Moreover, the coefficients for the interventions are almost all statistically significant at the 5 percent level, implying that the exclusion restrictions in the selection model are sufficiently strong. To check for multicollinearity between the independent variables and the inverse mills ratio, equation (1) is estimated using OLS with the Heckman-correction (i.e., inverse Mill’s ratio) added as an additional regressor (Supplementary Table A5.1). All variance inflation factor scores are around 1, hence we do not find evidence for multicollinearity (Supplementary Table A5.2). The output of the Ramsey Reset test reports a *p*-value of 0.33, meaning we do not find evidence for omitted variables (Supplementary Table A5.1). Since the test outputs provide no indication of present sample-induced bias, the instruments for ‘*Savings Account*’ (i.e., Asaan savings account opened with Meezan Bank) are strong, and additional checks do not report any violation of the assumptions, the OLS-regression (i.e., Eqn. 3) may be carried out.

The results from the OLS-regression are consistent with the results from the Heckman selection model: the output reports a positive association between the female client’s decision-making power over her Asaan savings account and her savings balance in that account (see Supplementary Table A6, Column 2). Still, the results of this OLS-regression should be interpreted with caution. As reported in Section “Sample Characteristics,” respondents that opened an Asaan savings account slightly differed in, e.g., age and literacy from the respondents who did not. Besides, the existence of influential omitted variables cannot be completely ruled out since decision-making power over the savings in the Asaan savings account was not randomized. Thus, sample-induced bias and omitted variable bias cannot be completely ruled out.

Considering the covariates, household income appears to be an important predictor of the total amount saved in the Asaan savings account. The coefficient of household income is positive and statistically significant at the 1 percent level for all three estimations: the Heckman selection model, the two-stage Heckit model, and the OLS regression. Further, the coefficient for having other formal savings is negative and statistically significant at the 10 percent level for the Heckman selection model (Table 3, Column 1), the two-stage Heckit model (Column 3) and the OLS regressions (Supplementary Table A6). Suggesting that the married female respondents with other formal savings save less in the Asaan savings account. The other control variables are not statistically significant.

### Robustness Tests

To test the robustness of the results, several additional tests are performed. First, we test the robustness of the results to the definition of *Control over the Asaan savings account*. In the previous models (Eqns. 1 and 3), ‘*Control*’ was defined as having full decision-making power or joint decision-making power with the husband and other household members. The dummy variable ‘*Control*’ equals ‘1’ if the participant has full or joint decision-making power, and equals ‘0’ if she has no decision-making power at all. In the following robustness regressions, we use an alternative specification for ‘*Control.*’ The alternative specification for ‘*Control*’ includes three categories: (i) the participant has no decision-making power (reference category), (ii) joint decision-making power, and (iii) full decision-making power over the use of the accrued savings in the account. The association of control with total savings is again tested with the Heckman selection model and an OLS-regression (Table 4A).

**TABLE 4A:** Robustness tests with total savings and No, Joint, and Full control.

Variables	(1) Heckman with clustered SEs		(2) OLS with clustered SEs		(3) OLS	
**S (total savings)**						
Joint_Control	2,242	(*p* = 0.106)	2,206	(*p* = 0.127)	2,206	(*p* = 0.075)
	(1,388)		(1,391)		(1235)	
Full_Control	5,742	(*p* = 0.078)	5,854	(*p* = 0.072)	5854	(*p* = 0.108)
	(3,254)		(3,099)		(3632)	
Age	25.18	(*p* = 0.737)	18.68	(*p* = 0.803)	18.68	(*p* = 0.739)
	(74.9)		(73.89)		(55.9)	
HH_Income	3,460	(*p* = 0.007)	3,444	(*p* = 0.015)	3,444	(*p* = 0.002)
	(1,293)		(1,301)		(1085)	
Read_Urdu	638.0	(*p* = 0.267)	716.2	(*p* = 0.222)	716.2	(*p* = 0.227)
	(575.0)		(570.2)		(592.5)	
HH_Size	−112.11	(*p* = 539)	−106.3	(*p* = 0.565)	−106.3	(*p* = 0.615)
	(182.6)		(182.0)		(211.4)	
Formal_Save	−3,421	(*p* = 0.109)	−3,129	(*p* = 0.132)	−3,129	(*p* = 0.077)
	(2135)		(1,998)		(1768)	
Constant	19,357	(*p* = 0.000)	20,773	(*p* = 0.000)	20,773	(*p* = 0.000)
	(4,460)		(3,674)		(2841)	
**Savings account**						
R_speech_Subsidy_Help	1.871	(*p* = 0.000)				
	(0.206)					
R_speech_Subsidy	1.114	(*p* = 0.000)				
	(0.199)					
R_speech_Help	0.657	(*p* = 0.000)				
	(0.176)					
R_speech	0.427	(*p* = 0.081)				
	(0.245)					
C_speech_Subsidy_Help	0.630	(*p* = 0.000)				
	(0.082)					
C_speech_Subsidy	0.278	(*p* = 0.003)				
	(0.245)					
C_speech_Help	0.348	(*p* = 0.024)				
	(0.082)					
Age	0.011	(*p* = 0.018)				
	(0.004)					
HH_Income	0.0248	(*p* = 0.732)				
	(0.073)					
Read_Urdu	−0.019	(*p* = 0.598)				
	(0.037)					
HH_Size	0.012	(*p* = 0.454)				
	(0.016)					
Formal_Save	−0.236	(*p* = 0.123)				
	(0.153)					
Constant	−1.751	(*p* = 0.000)				
	(0.233)					
Rho	0.111	(*p* = 0.400)				
	(0.131)					
Observations	1786		512		512	
*R* ^2^			0.04		0.04	

*This table reports the output of the estimations with dummies for No, Joint, and Full control instead of the variable Control, including two groups: (i) having no control, and (ii) at least joint control. R_speech, religious speech. C_speech, conventional speech. Subsidy, receipt of subsidy for opening the account. Help, assistance with filling in application forms. In Columns 1 and 2, the standard errors are clustered on the branch level.*

Similar to the results of the previous Heckman selection model estimation, the results of the robustness Heckman estimation indicate that there is a statistically significant positive association between control and total savings in the Asaan savings account (see Table 4A, Column 1). Both the respondents with joint control and the respondents with full control have a statistically significantly higher closing balance than respondents without control in May at the 10 percent level. Moreover, the coefficient of the respondents with full control is higher than the coefficient of respondents who have joint control. In other words, the married women with full control have on average a higher closing balance/savings in the account than the married women with joint control, as can be intuitively expected. However, this difference in total savings between respondents with joint and with full control is not – but close to being – significant at the 10 percent level. Note that the number of respondents with full control is quite low. With more statistical power, this difference would most likely have been statistically significant. Further, the output of the test of independent equations suggests that there is no sampling-induced bias.

The OLS-regressions with and without clustered standard errors on branch code report similar results (see Table 4A, Column 2). In the OLS-regressions without clustered standard errors, the coefficient for joint control and the coefficient for full control are both significant at the 10 percent level. The *p*-values equal 0.08 and 0.10, respectively. In the OLS-regressions with clustered standard errors, the coefficient for joint control is almost statistically significant at the 10 percent level (*p*-value equals 0.127). The coefficient for full control is statistically significant at the 10 percent level (*p*-value equals 0.072). Hence, the output obtained from the robustness OLS-estimations are also in line with the previously stated results.

Secondly, we test the robustness of the results to the specification of the dependent variable. In this robustness test, we use the closing balance in April instead of the closing balance in May. The output of both the Heckman selection model and OLS-regression hold similar results and are therefore suggesting the results documented in Table 3 are not unique to the month of May (see Table 4B).

**TABLE 4B:** Robustness tests with total savings in April and Control.

Variables	(1) Heckman selection model with clustered SEs		(2) OLS with clustered SEs		(3) OLS	
**S (total savings in April)**						
Control	2,013	(*p* = 0.099)	1,988	(*p* = 0.117)	1,989	(*p* = 0.070)
	(1219)		(1,218)		(1,097)	
Age	24.06	(*p* = 0.713)	19.18	(*p* = 0.769)	19.18	(*p* = 0.698)
	(65.51)		(64.61)		(49.4)	
HH_Income	3,002	(*p* = 0.008)	2,992	(*p* = 0.015)	2,992	(*p* = 0.002)
	(1131)		(1133)		(963.1)	
Read_Urdu	619.3	(*p* = 0.244)	680.9	(*p* = 0.214)	680.9	(*p* = 0.197)
	(531.4)		(532.4)		(527.5)	
HH_Size	−74.33	(*p* = 0.655)	−69.49	(*p* = 0.680)	−69.49	(*p* = 0.712)
	(166.1)		(166.0)		(188.1)	
Formal_Save	−2,717	(*p* = 0.147)	−2,480	(*p* = 0.173)	−2,480	(*p* = 0,144)
	(3922)		(1,759)		(1,566)	
Constant	16,706	(*p* = 0.000)	17,802	(*p* = 0.000)	17,802	(*p* = 0.000)
	(3922)		(3,230)		(2509)	
**Savings account**						
R_speech_Subsidy_Help	1.873	(*p* = 0.000)				
	(0.205)					
R_speech_Subsidy	1.114	(*p* = 0.000)				
	(0.199)					
R_speech_Help	0.656	(*p* = 0.000)				
	(0.175)					
R_speech	0.426	(*p* = 0.082)				
	(0.244)					
C_speech_Subsidy_Help	0.630	(*p* = 0.000)				
	(0.081)					
C_speech_Subsidy	0.281	(*p* = 0.003)				
	(0.093)					
C_speech_Help	0.351	(*p* = 0.022)				
	(0.004)					
Age	0.011	(*p* = 0.018)				
	(0.004)					
HH_Income	0.025	(*p* = 0.733)				
	(0.073)					
Read_Urdu	−0.020	(*p* = 0.595)				
	(0.037)					
HH_Size	0.012	(*p* = 0.452)				
	(0.016)					
Formal_Save	−0.236	(*p* = 0.122)				
	(0.153)					
Constant	−1.751	(*p* = 0.000)				
	(0.232)					
Rho	0.097	(*p* = 0.460)				
	(0.131)					
Observations	1786		512		512	
*R* ^2^			0.04		0.04	

*This table reports the output of the estimations with total savings in April (i.e., the saving closing balance in April) as the outcome variable, instead of ‘Total savings at the end of May’ (i.e., the saving closing balance in May). R_speech, religious speech. C_speech, conventional speech. Subsidy, receipt of subsidy for opening the account. Help, assistance with filling in application forms. In Columns 1 and 2, the standard errors are clustered on the branch level.*

## Discussion

### Main Findings

The purpose of this study is to examine the association between a woman’s perception of her decision-making power over a micro-savings account and the amount saved in this account, in a setting with high gender inequality. The expectation is that female clients, who perceive to have at least joint control over their formal savings account, save more in this account, compared to female clients who do not. To test for this association and correct for potential sample-induced bias, we estimate a Heckman-selection model and conduct several robustness analyses.

First, we find that, out of the 512 married Pakistani women who opened an account, 2 percent indicated to have full control over the opened bank account, 77 percent indicated to have joint decision-making power, and 21 percent indicated not to have any control. Next, the results of the Heckman selection model estimation report that women with at least joint control over the account saved on average 2,339 PKR (22.40 USD) more in the first 8 months after the intervention than women who perceive to have no control. This result appears to be robust to the exact month used for total savings and the specification of control. The found difference in savings is considerable, taking into account that (i) most households earn a combined income below the 2015 upper-middle income poverty line of 5,624 PKR per adult per month^13^ (World Bank, 2020), and (ii) women are seldomly the main income earners. In other words, over a period of 8 months, women with control over the account save roughly half a month of a middle-class person’s income more than women without control. Hence, the results of this study provide evidence for the positive association between control over the account and the level of savings in the account.

Considering the slopes of the graphs in Figure 1 and the negligible difference in the average first deposit amount across control, one could conclude that the difference in account balances levels is slowly growing over time. Participants with control over savings deposit slightly higher amounts, and subsequently receive slightly higher profits from the Mudarabah arrangement (see Supplementary Figure A7 and Supplementary Tables A7, 8). The responses to additional survey questions hint that the higher deposits for respondents with control over the account could be partially due to higher substitution of other saving sources with the Asaan Savings Account among this group. Specifically, the share of respondents that indicated to save less in other sources as a result of opening the account is larger for the group with control (see Supplementary Tables A9A,B). To the question inquiring in what sources they saved less, savings in cash were indicated more often among respondents with control over the account.

Our results add to the findings of Anderson and Baland (2002), and Schaner (2016), who emphasize the role of control over savings in women’s decisions where and how much to save. As in Schaner (2016), we find that women with more control over the savings account save more in that account. However, unlike Schaner (2016), the degree of control in our study is not exogenously varied, but applies to women who have all opened savings accounts whose design in principle offers maximum degree of control. Anderson and Baland (2002) assert that wives try to protect their savings against claims of their husband and other household members by participating in informal saving clubs. The responses in our survey questions hint at a similar behavior, as mostly women with control over their account appear to partially substitute money investments in other sources for savings in their Meezan Bank account. To the question which sources are invested in less, savings in cash – which are known to be highly sensitive to claims of others (Ashraf et al., 2006; Banerjee and Mullainathan, 2010; Dupas and Robinson, 2013b) – were checked most often by respondents with control over their bank account. Whereas much fewer women with such control seem to for instance partially substitute savings in their informal saving clubs with money in their bank account. Hence, our study contributes to the academic literature by documenting that also in the decision whether to use formal saving mechanisms registered in solely their name, women consider their control over this mechanism. Still, this study is among few academic studies that explicitly focus on the female account holder’s decision-making power over savings in her single-owned formal account within a setting with severely wide gender-gaps and prevalent gender norms. More empirical evidence is needed to better understand this relationship and what context specific factors might influence the strength of this association.

### Limitations

One important limitation of this study is that the variable capturing the decision-making power is relatively narrow. The variable we use is based on only one survey question, asking whether the female client can decide how to use the money accrued in the account by herself, jointly or not. There might be still some variance in the level of decision-making power within the category joint control. One could for instance have an equal control over the money relative to her husband or household members, or a minority or majority control. In other words, the female client may decide on how to use a specified share of the money in the account, agreed upon with the others involved.

An important area for further research encompasses the determinants and dimensions of control over financial products. When these dimensions are uncovered, one could construct a latent variable or instruments that capture the decision-making power over the provided bank account more accurately. With such variable the relationship between decision-making power over this bank account and usage could be estimated more precisely.

Another limitation is the self-reported nature of the variable ‘Control,’ in the context that women might prefer not to report lack of control over the account they experience. For instance, they could (i) be concerned that the study team, Akhuwat, Meezan Bank preferred to hear that they have control over the account, or (ii) be afraid that their husband found out about their response and report control over the account even if it was not true. However, we are not overly concerned about this matter in the context of our study for the following reasons: First, we implemented a transparent and thorough consent process during which we assured the participants about the confidentiality of their answers. As the follow-up survey (which asked them about the degree of control) was conducted one-on-one at a mutually agreed upon time and location (before visiting the participants at their homes or workplace, we set-up an appointment with them over the telephone) that guaranteed privacy (in most cases, women were interviewed at home and a family member was present in the dwelling, though it was rarely the husband and though we made sure that this family member would not be able to listen in on the interview process), we think it is unlikely that the women felt pressured to not report (or report, for that matter) lack of control.

Relatedly, since the relevant question was part of the follow-up survey (which came a few months after the baseline survey), some degree of rapport and trust regarding the confidentiality of their responses had been established between the research team and the interviewee by the time the question was asked. Importantly in this context, the enumerators who conducted the survey were neither staff of Akhuwat nor staff of Meezan bank but our own trained field staff. Hence, they are very likely to have been perceived as “neutral” regarding this matter by the respondents.

Second, note that in our broader intervention, the savings account was not promoted in a women’s empowerment context or in the context of increasing women’s control over financial resources. In fact, we promoted this account to both men and women, and we only focus on the sample of married women in the present paper because of the specific question we are investigating. Thus, the women in our sample should not have gained the impression that our study team or Akhuwat or Meezan bank had a particular interest in them exerting a high degree of control over the account, and it is not ex ante clear why the husbands, in the conservative social context of Pakistan, would necessarily prefer that their spouses reported control over their account.

Third, note that while the account was by default operated singly, the women had the option of nominating a person to operate the account on their behalf. In fact, it is not unusual or socially unacceptable in Pakistan for that to be the case. Hence, we do not think that the women faced a binding constraint in terms of social norms to actually report having little control over the account.

Last but not least, it is worth noting that we are likely to be underestimating the magnitude of our effect of interest if women overstate the degree of control. In the latter case, some women who exert no control over their accounts would end up being represented in the group which reported control. If indeed control has a positive effect on savings, then overreporting of control is likely to lead to an underestimate of the effect of control on savings.

A final limitation is that we cannot fully rule out potential omitted variables. Comparing the group of female clients with at least some control and no control, there were no significant differences between baseline characteristics such as household size, number of sons and level of education (Table 2). The output of the Ramsey Reset test indicates the null hypothesis of no omitted variables cannot be rejected (*p*-value = 0.33) (Supplementary Table A5.1), i.e., indicates our analysis is not subject to omitted variables. Still, we cannot fully dismiss that the difference in the savings level is determined by another unobserved characteristic correlated with female client’s control over the account, as control was not randomized. Therefore, we should interpret the coefficient size with some caution.

### Going Forward

Considering policy implications, this study highlights the importance of recognizing that product design alone cannot offer a satisfactory solution when it comes to supporting women in exercising control over bank accounts. To advance the Agenda for Sustainable Development in terms of poverty reduction among women and addressing gender inequalities through the financial inclusion of women, funders and suppliers of formal saving services need to take the impacts of gender norms and associated low bargaining power on the usage of banking services by women into account in a more holistic way. In highly gender unequal contexts, the implicit assumptions that (i) women who have opened a bank account will start using it regularly in this account and, (ii) that the savings that accrue in these accounts will ultimately improve women empowerment, are less likely to hold for women who are less empowered to begin with. This can even be observed when the account is single-operated, obtaining an ATM card is costly and photo-IDs are needed for transactions at the bank branch: Only 2 percent of the women who opened a bank account indicate to have full decision-making power over their account and women without any decision-making power save substantially less *via* these accounts.

Other remedies that emerge from the literature, e.g., Ashraf (2009), is to limit the observability of women’s savings to any other parties that may lay claim to them, such as husbands and other family members. However, such an approach may not be feasible in contexts such as ours, where the effectiveness of providing women with a bank account whose existence or contents can be kept private, may be hampered by women having limited mobility outside of the household/immediate neighborhood, and low literacy levels.

More research is needed on factors determining women’s control over formal bank accounts. Knowing the determinants could provide policy makers with a better understanding on how to make accounts more useful especially to those women who are least empowered to begin with. Hence, such knowledge would enable financial policy makers to improve the effectiveness and efficiency of their efforts to advance women empowerment through financial inclusion.

Interestingly, to the survey question inquiring participants’ satisfaction with how willing customer service representatives were to listen and respond to their needs, a larger share of women with control indicated to be satisfied compared to women without control (see Supplementary Table A10). Staff from formal financial institutions might have (conscious or unconscious) biases about women and their need for financial services. Hence, it would be worthwhile to for instance study the effects of not only workshops designed to challenge gender norms and empower women provided to the account holders and their husbands, but also to study the effects of training and instruction programs for frontline staff that focus on how to treat and provide services to illiterate, female customers. In such highly gender inequal contexts, recruitment of female agents and female financial policy makers might be challenging due to the existing norms, but highly awarding in the long run. Hence, another interesting area to study includes the effects of female employees on women’s account use. In short, an important avenue for future research includes the determinants of women’s control over financial services offered to them that go beyond product design.

In summary, increased financial inclusion could help many women in escaping the poverty trap. However, to advance financial inclusion, funders and policy makers must recognize that in highly gender inequal contexts, their female customers may not be in (full) control of even single-owned bank accounts, and subsequently use it less. Future research and policy efforts should be directed at understanding the underlying factors of women’s control over their accounts and how to best address these, in order to develop successful policies that increase female client’s account use. Only then, financial inclusion could advance women’s empowerment of especially those women who are least empowered to begin with.

## Data Availability Statement

The original contributions presented in the study are publicly available. The data and additional material supporting the conclusions of this article can be found here: https://doi.org/10.34894/JNEB8U, DataverseNL, V1.

## Ethics Statement

The studies involving human participants were reviewed and approved by the Research Ethics Committee of the Faculty of Economics and Business at the University of Groningen (Approval Number 2017-10-06 ECFEB), Akhuwat and Meezan Bank. The patients/participants provided their written informed consent to participate in this study.

## Author Contributions

ED and RL contributed to the conception and design of the study. SA organized the database. ED performed the statistical analysis and wrote the first draft of the manuscript with contributions from RL and AM. AM and ED revised the manuscript with inputs from RL and SA. All authors have read, and approved the submitted version.

## Conflict of Interest

The authors declare that the research was conducted in the absence of any commercial or financial relationships that could be construed as a potential conflict of interest.

## Publisher’s Note

All claims expressed in this article are solely those of the authors and do not necessarily represent those of their affiliated organizations, or those of the publisher, the editors and the reviewers. Any product that may be evaluated in this article, or claim that may be made by its manufacturer, is not guaranteed or endorsed by the publisher.

## Footnotes

1 Research by the Food and Agriculture Organization (FAO, 2020) for instance documents that women are at a greater risk of malnutrition than men in households, which are susceptible to food insecurity.

2 In particular, such accounts are predicted to enhance women’s ability to refuse money demands from others, as, e.g., a third party is involved, formal permission is needed for others wanting to access the money, and a withdrawal fee might need to be paid (Schaner, 2016).

3 In fact, it might well be that the same or similar norms to the ones, which contribute to a lack of women empowerment in the first place, also affect womens’ account usage. Examples of such norms comprise “women should not have financial privacy from their husband” and “women should not have savings on their own” (Koning et al., 2021).

4 In 2015, if a person earned more than 184.9 PKR (i.e., US$ 5.50, 2011 PPP) per day, they were categorized as belonging to either the upper middle-income class or the high-income class.

5 Note that the average household size exceeds 7 for our sample.

6 Examples of financial assets are formal and informal savings. Other types of assets frequently discussed in the context of women empowerment include real estate, vehicles, agricultural land, equipment and livestock, business equipment, human capital, and social capital (i.e., membership in groups and organizations) (Meinzen-Dick et al., 2011).

7 For the photo account holders a judiciary note was required to obtain an ATM card. The price of the ATM card was approximately 14 times higher than the check book price at the time of the interventions. Of the 513 married women that opened an account, only 20 have an ATM card (see Supplementary Table A1).

8 The decision to only include married women may to some extent result in sample selection bias. Nevertheless, the groups ‘men’ and ‘unmarried women’ are very small relative to ‘married women’, hence the risk of sample selection bias is rather limited.

9 Using the 2017 average exchange rate of 1 USD: 104 PKR. Retrieved from: https://www.poundsterlinglive.com/best-exchange-rates/best-us-dollar-to-pakistani-rupee-history-2017.

10 Note that Akhuwat’s Family Enteprise Loans are also provided for start-ups.

11 These six married, Christian women did not open an account with Meezan bank.

12 Since our sample includes only married female participants, it seems sufficiently safe to assume that there are no participants who jointly decide over the use of savings with household members other than their husband only. In other words, the category ‘no control’ most likely only incorporates participants who have no decision-making power over the use at all.

13 The upper middle income class poverty line equals 184.9 PKR in 2015 or US$ 5.50 (2011 PPP) per day per capita.
