# Supplementary material for: Trapped by the Lack of Control Over Savings: Evidence From Pakistan
Source: Front Psychol. 2022 May 18;13:867841. doi: 10.3389/fpsyg.2022.867841 (PMC9161557; doi:10.3389/fpsyg.2022.867841)
Supplement: Supplementary file 1 [file Data_Sheet_1.docx]

Appendix

**Section A1: ATM-access by the level of Control**

**Table A1: Distribution of Meezan Bank ATM-cards across control over the savings account**

|  | **No control** | **Joint control** | **Full control** |
| --- | --- | --- | --- |
| **Not in possession of an MB ATM-card** | 103 | 370 | 11 |
| **In possession of an MB ATM-card** | 4 | 16 | 0 |

**Section A2: Study design**

**Figure A2: Flowchart diagram – Treatment arms**

29 participants dropped out of 1,094 potential participants

46 participants dropped out of 1,126 eligible participants

**Notes:** Figure A2 is a modified version of *‘Figure A1: Flowchart diagram for treatment arms’* from Ahmad, Lensink, and Mueller (2020). The diagram shows the experimental design and randomization procedure (from left to right). First, Ahmad, Lensink, and Mueller (2020) randomly assign the type of speech – religious or conventional – at branch level. Second, in each stratum, the researchers randomize provision of subsidized access to Islamic savings accounts at the individual level. Third, the researchers randomize (at the individual level) assistance with filling the account opening form within each group.

**Section A3: Variable description**

**Table A3: Specification of variables**

| **Variable Name** | **Question** | **Coding of Responses** | **Source** |
| --- | --- | --- | --- |
| **Age** | Date of birth of borrower surveyed | DD/MM/YYYY………………..Age in years | Baseline survey |
| **HH_size** | How many other people – not including yourself – live in your household? | ___ members……….…………..Number of  hh members | Baseline survey |
| **N_Sons** | How many sons/daughters do you have? | ___ sons…………………..…….Number of  Sons | Endline survey |
| **HH_Income** | What is your overall household income? | Less than 30,000 PKR……………………..0  More than 30,000 PKR………….................1 | Baseline survey |
| **Borrower_Income** | On average, how much income do you personally earn in a month? | Less than 10,000 PKR……………………..0  10,000 to 20,000 PKR…………………..…1  > 20,000 PKR………………………...……2 | Baseline survey |
| **Main_Earner** | Who in your household earns the majority of income, excluding you? | No one besides me…………………….…...1  Spouse……………………………….……..1  Father (in-law)……………………………..0  Mother (in-law)………………………..…..0  Other………………………………….……0 | Baseline survey |
| **Read_Urdu** | Are you able to read in Urdu, e.g., a letter? | No………………………………………….0  Yes, with difficulty……………………...…1  Yes, easily………………………………….2 | Baseline survey |
| **Grades_Passed** | What is the highest grade of (government) schooling you successfully completed? | __ grade…………………………..Grade level  as integer | Baseline survey |
| **First_Loan** | First loan from Akhuwat? | Yes………………………………………....1  No…………………………….……………0 | Administrative data |
| **Formal_Save** | Do you have any of the following formal forms of savings? | Money on Mobile account……………….. 1  Money on National Savings Account…..…1  Money in Government Saving Certificates………………………………...1  Savings, special savings, regular income savings……………………………………..1  Prize Bonds………………………………..1  None of the above………………………....0 | Baseline survey |
| **Self_Empl** | How would you describe the type of work that generates the majority of your income? | Self-employment…………………………..1  Other………………………………….……0 | Baseline survey |
| **Not_Work** | How would you describe the type of work that generates the majority of your income? | Not working……………………………….1  Other………………………………………0 | Baseline survey |
| **Control** | Can you, by yourself, decide how to use the money that accrues in the bank account? | Yes……………………………………..….1  No, together with my spouse……………...1  No, together with my spouse and in-laws…………………………………….….1  No (but different from previous scenarios)………………………………..…0 | Follow-up survey |
| **No Control** | Can you, by yourself, decide how to use the money that accrues in the bank account? | Yes……………………………………..….0  No, together with my spouse……………...0  No, together with my spouse and in-laws…………………………………….….0  No (but different from previous scenarios)……………………………….…1 | Follow-up survey |
| **Joint Control** | Can you, by yourself, decide how to use the money that accrues in the bank account? | Yes……………………………………..….0  No, together with my spouse……………...1  No, together with my spouse and in-laws…………………………………….….1  No (but different from previous scenarios)………………………………..…0 | Follow-up survey |
| **Full Control** | Can you, by yourself, decide how to use the money that accrues in the bank account? | Yes……………………………………..….1  No, together with my spouse……………...0  No, together with my spouse and in-laws…………………………………….….0  No (but different from previous scenarios)………………………………..…0 | Follow-up survey |
| **Total savings in the Asaan savings account** | The closing balance of the Asaan savings account (from Meezan Bank) at the end of May, 2018. | __ PKR | Endline survey or administrative data of Meezan Bank |
| **Total amount of deposits (March to May)** | The total amount deposited in the Asaan savings account from the 1^st^ of March to the end of May. | __ PKR | Endline survey/  administrative data of Meezan Bank |
| **Total received profit/Mudarabah (March to May)** | The total amount of Mudarabah received in the Asaan savings account from the 1^st^ of March to the end of May. | __ PKR | Endline survey/  administrative data of Meezan Bank |

**Section A4: Empirical strategy**

**A4: Heckman-selection model**

In this study, we test for the association between the female owner’s control and the amount of savings in the account. Naturally, the dependent variable of interest – ‘Total savings in the Meezan Bank account’ – is only observed for participants that open an account. The independent variable of interest – ‘Control over the savings in the Meezan Bank account’ – is likewise limited to the participants who opened a bank account. Data for the covariates are collected for all 1,978 married female participants.^[[1]](#footnote-1)^ Hence, the data is incidentally truncated.

Since the opening of the account itself was not randomized – but the encouragements to open an account were –, the sample of account openers is self-selected. Such self-selection could have resulted in differences between the group of account openers and the group of individuals who did not open an account, hence in an unrepresentative sample. For instance, literate women may be more inclined to open an account than non-literate women. Using a simple ordinary least squares (OLS) estimation based on solely the subsample of account openers may therefore lead to coefficients that suffer from sample selection bias.

To investigate potential selection sampling selection bias, induced by potential self-selection of individuals for opening the account, we implement a Heckman-selection model. The Heckman-selection model consists of the model’s primary estimation specification and of a selection specification. The latter represents the sample selection process. Based on the selection specification, the Heckman selection-model will calculate a correction for the differences between groups (account-openers and non-openers). This correction will be incorporated in the primary estimation. When this correction-term is of considerable size (i.e., statistically significant), there is a sampling selection problem and the primary estimation specification should be estimated with this Heckman-correction. When the correction-term is of negligible size (i.e., not statistically significant), the results indicate that there is no evidence for a sampling-selection problem. In the latter case, the primary estimation specification could be estimated without this Heckman-correction using a simple OLS.

Our model’s primary estimation specification (eqn. 1) and selection specification (eqn. 2) are as follows:

$S_{i}^{*}= x_{1i}^{'}\beta_{1}+\varepsilon_{1i}$, (eqn. 1)

where $S_{i}^{*}= \left\{ \begin{aligned} S_{i}^{*} if {Savings account}_{i}=1 \\ - if {Savings account}_{i}=0 \end{aligned} \right.$

${Savings account}_{i}^{*}=x_{2i}^{'}\beta_{2}+\varepsilon_{2i}$, (eqn. 2)

${\mathrm{where}Savings account}_{i}=\left\{ \begin{aligned} 1 if {Savings account}_{i}^{*}>0 \\ 0 if {Savings account}_{i}^{*}\leq0 \end{aligned} \right.$

The error terms are distributed according to:

$\left[ \begin{matrix} \varepsilon_{1i} \\ \varepsilon_{2i} \end{matrix} \right]\sim N \left( \left[ \begin{matrix} 0 \\ 0 \end{matrix} \right] , \left[ \begin{matrix} \sigma^{2} & \sigma_{12} \\ \sigma_{12} & 1 \end{matrix} \right] \right)$, (eqn. 3)

where$\sigma_{12}=Cov \left( \varepsilon_{1i}, \varepsilon_{2i} \right)=\rho*\sigma.$

Here, $S_{i}^{*}$is the savings balance in the Asaan savings account at the end of May, 2018, ${Savings account}_{i}^{*}$ represents whether the respondent opened the Asaan savings account, and $\rho$ is the correlation between the error terms $\varepsilon_{1i}$ and $\varepsilon_{2i}$.

In the primary specification, $S_{i}^{*}$, i.e., ‘Total savings in the Meezan Bank account’, is defined as the closing balance in May; approximately 8 months after the loan disbursement meetings. Data on participant’s monthly savings balances was collected from Meezan Bank’s administrative data and the participants check (log)books during the endline survey. The participant needs this check (log)books for making withdrawals and payments.

Next, $x_{1i}^{'}$ includes the independent variable of interest – *Control over the savings in the Asaan savings account* – and control variables. The variable ‘*Control’* is binary; indicating ‘0’ if the respondent has no control over the savings and indicating ‘1’ if the respondent has at least some control over the savings. 79 percent of respondents has joint or full control, and 21 percent has no control over how the savings accrued in the account are used. Some control implies that the respondent decides over the use of the accrued savings i) on their own, ii) together with her husband or iii) together with her husband and other household members.^[[2]](#footnote-2)^ As mentioned in section 2.2, the variable ‘*Control’* is based on self-reported data, collected approximately three months after the loan disbursement meetings took place.

Besides, $x_{1i}^{'}$ includes the following set of control variables: age, household income, household size and ability to read. For household income, a dummy-variable is included, which equals one if the household income is above 30,000 PKR and equals zero if below 30,000 PKR. Household size is defined as the number of people (excluding the respondent) living in your household. Ability to read is captured by an ordinal variable, which is equal to ‘2’ if the participant indicates to be able to easily read in Urdu, ‘1’ if the participant indicates to be able to read in Urdu with difficulty, and ‘0’ if the participant is not able to read in Urdu.

In the selection specification (eqn. 2), the dummy *‘Savings account’* equals ‘1’ if the participant opened an Asaan savings account with Meezan Bank after the intervention and ‘0’ if the participant did not. The $x_{2i}^{'}$ includes dummies for the encouragements provided in the RCT: receipt of a subsidy (‘*Subsidy’),* assistance with completing the application form (‘Help’) and a religious speech (‘R_speech’). The treatment dummies make good instruments since the treatments’ objective was to increasing uptake, their coefficients are positive and statistically significant, and the treatment selection was random (see Ahmad, Lensink, and Mueller; 2020). Hence, these instruments are quite strong, exogenous, and only indirectly affect the level of savings via uptake. See Figure A2 (in the appendix) for the specification of the treatment variables. Furthermore, the $x_{2i}^{'}$ includes covariates for age, household income, household size and the ability to read in Urdu. The data for the covariates are all collected as part of the baseline survey.

The primary specification is estimated using a Heckman-selection model with a Full Information Maximum Likelihood estimator (FIML) and a Heckit-selection model (two-step procedure) with a Limited Information Maximum Likelihood (LIML). The main differences between the two types of Heck-man selection models, is that the first assumes that $\varepsilon_{1i} and \varepsilon_{2i}$ follow a bivariate normal distribution (see eq. 3). When this assumption holds, the FIML produces a more efficient estimator than the Heckman two-step procedure with a Limited Information Maximum Likelihood estimator. In the two-step procedure, the Inverse Mills ratio (λ) is computed to check for the potential sample selection issue. When the assumption concerning the bivariate normal distribution of the errors does not hold, LIML would be preferable. Both the FIML and LIML estimations are carried out. Since the corresponding coefficients and p-values only slightly differ, the assumption on the error distribution does not appear to be violated and the FIML is therefore preferred over the LIML for the primary analysis.

To test for selection bias due to the incidental truncation, test of independent equations with a χ^2^-distribution is used. The null hypothesis of this test states that the correlation between $\varepsilon_{1i}$ and $\varepsilon_{2i}$ (ρ) is equal to zero. If this null hypothesis is rejected, sample-induced selection bias due to the incidental truncation is likely. If the null hypothesis cannot be rejected and assumptions – e.g., having a sufficiently large sample size, no multicollinearity between the x-variables and the ‘inverse Mills ratio’,^[[3]](#footnote-3)^ and no important omitted variables – are satisfied, the selection bias is negligible.

The output of the Heckman-selection model estimations provides a correction-term of negligible size. Specifically, in the first Heckman-selection model estimation, $\rho$ (i.e., the correlation between the error terms $\varepsilon_{1i}$ and $\varepsilon_{2i}$) is not statistically significant. In the two-step Heckman-selection model estimation (i.e., the Heckit model), the so-called inverse Mills ratio^3^ (i.e., the correction term) is also statistically insignificant. Thus, the two types of Heckman selection models do not provide evidence for sample selection bias. This allows us to estimate the primary estimation specification without the correction term, using ordinary least squares.

**Section A5: Multicollinearity check**

**Table A5.1: Association Total savings and Control – OLS with Mills-correction estimation**

| **VARIABLES** | **Coefficients &** | **p-values** |
| --- | --- | --- |
|  | **Std. Errors** |  |
| **S (Total savings)** |  |  |
| **Control** | 2,336.9 | (p=0.058) |
|  | (1232) |  |
| **Age** | 31.7 | (p=0.570) |
|  | (55.82) |  |
| **HH_Income** | 3,552 | (p=0.001) |
|  | (1082) |  |
| **Read_Urdu** | 643.8 | (p=0.282) |
|  | (597.8) |  |
| **HH_Size** | -107.1 | (p=0.613) |
|  | (211.5) |  |
| **Formal_Save** | -3214 | (p=0.071) |
|  | (1779) |  |
| **Mills** | 1,224.8 | (p=0.304) |
|  | (1191) |  |
| **Constant** |  |  |
|  |  |  |
| **Observations** | 512 |  |
| **Adjusted R^2^** | 0.03 |  |
| **Ramsey RESET test F (3, 501)** | 1.17 | (p = 0.3216) |
| **Notes:** This table reports the primary model estimation (eqn. 1) of the two-step Heckman model (i.e., Heckit model), where instead of using STATA’s ‘Heckman twostep’ command, we estimate a probit-estimation for eqn. 2, calculate the Inverse Mills Ratio, and then carry out the regression for eqn. 1. Conducting this estimation allows us to check for multicollinearity. The results are almost identical to the output of the estimation using STATA’s Heckman two step command. | | |

**Table A5.2: Multicollinearity check – OLS with Mills-correction estimation**

| **VARIABLES** | **VIF** |
| --- | --- |
|  |  |
| **Control** | 1.00 |
| **Age** | 1.02 |
| **HH_Income** | 1.03 |
| **Read_Urdu** | 1.04 |
| **HH_Size** | 1.01 |
| **Formal_Save** | 1.03 |
| **Mills** | 1.05 |
| **Mean VIF** | 1.03 |
| **Notes:** This table reports the Variance Inflation Factor (VIF) of the primary model estimation including the Heckman-correction (i.e., the Inverse Mills Ratio). | |

**Section A6: Estimations of the primary model excluding the Heckman-correction**

**Table A6: Association Total savings and Control – OLS estimations**

| **VARIABLES** | **(1)** | **(2)** | **(3)** |
| --- | --- | --- | --- |
|  |  |  |  |
| ***S (Total savings****)* |  |  |  |
| **Control** | 2,512 | 2,307 | 2,307 |
|  | (p=0.044) | (p = 0.062) | (p = 0.114) |
| **Age** |  | 25.7 | 25.74 |
|  |  | (p = 0.643) | (p = 0.720) |
| **HH_Income** |  | 3528 | 3528 |
|  |  | (p = 0.001) | (p = 0.014) |
| **Read_Urdu** |  | 725.4 | 725.4 |
|  |  | (p = 0.221) | (p = 0.219) |
| **HH_Size** |  | -99.38 | -99.4 |
|  |  | (p = 0.638) | (p = 0.588) |
| **Formal_Save** |  | -2,939 | -2,939 |
|  |  | (p = 0.095) | (p = 0.156) |
| **Constant** | 22,087 | 20,390 | 20,390 |
|  | (p = 0.000) | (p = 0.000) | (p = 0.000) |
| **Observations** | 512 | 512 | 512 |
| **Adjusted R^2^** | 0.01 | 0.03 | 0.04 |
| **Notes:** This table reports the OLS estimation output of the primary model (eqn. 1) without the Heck-man correction term. In Column 1, the primary model is estimated without any covariates. In Column 2, the primary model including covariates is estimated. In Column 3, the primary model including covariates is estimated, with standard errors clustered at the branch level. | | | |

**Section 7: The association between amount deposited and level of Control**

**Figure A7: Monthly amount deposited in the account by the level of Control**

**
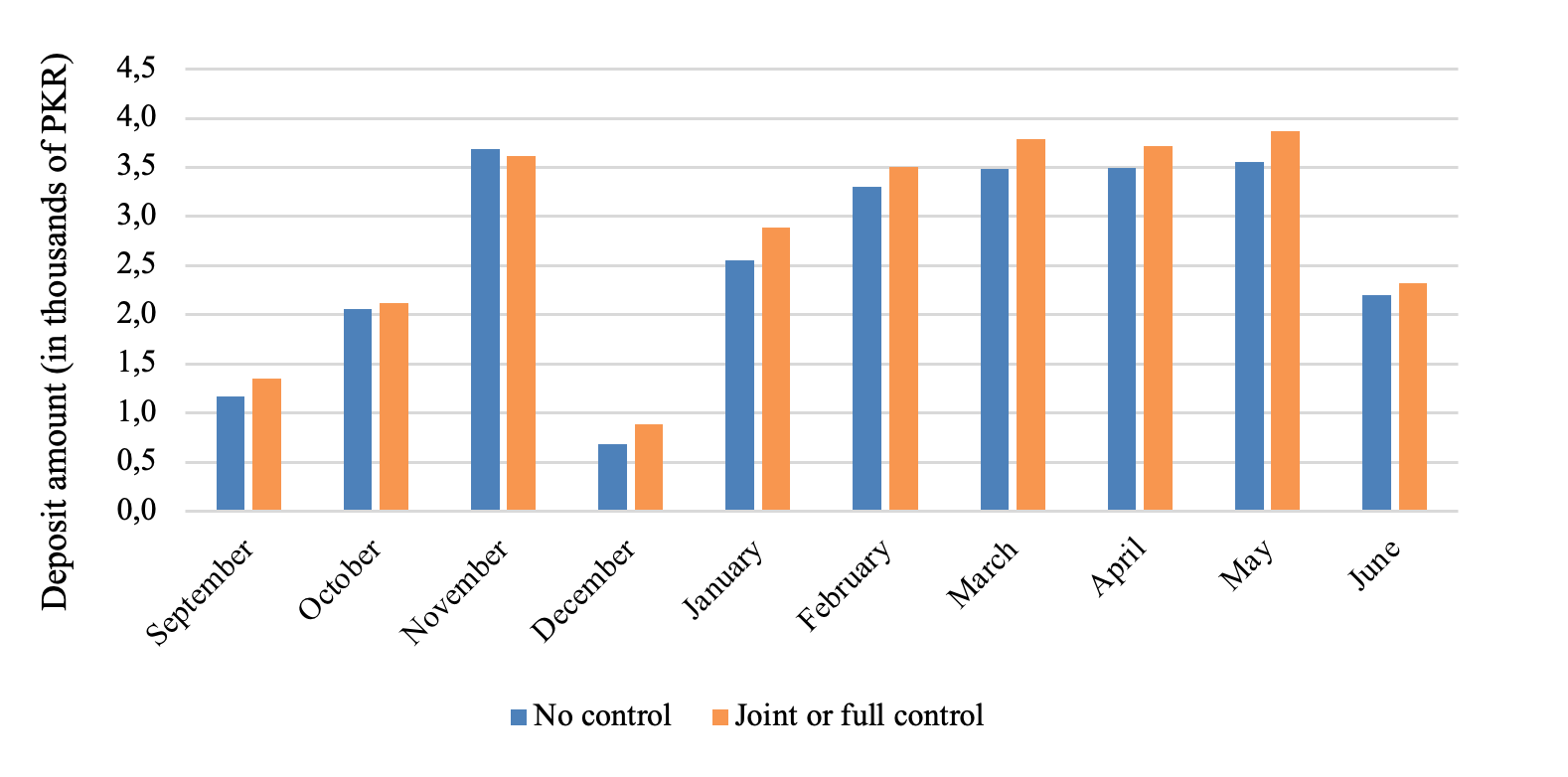
**

**Notes:** In the months of September and October, the number of accounts with at least one deposit was 67 and 254, respectively. In the months of November and December, this number was 280; in January and February, it was 396; and from March onwards, it was 512.

**Table A7: Amount of savings deposited and Control – Heckman selection model**

| **VARIABLES** | **Heckman selection model** | | | **Heckman selection model**  **(No, Joint, and Full control)** | |
| --- | --- | --- | --- | --- | --- |
| ***Total amount of deposits***  ***(March to May)*** | |  |  | |  |
| **Control** | 775.5 | p = 0.121 |  | |  |
|  | (500.1) |  |  | |  |
| **Joint Control** |  |  | 711.9 | | p = 0.155 |
|  |  |  | (500.4) | |  |
| **Full Control** |  |  | 2,988 | | p = 0.042 |
|  |  |  | (1471) | |  |
| **Age** | 11.0 | p = 0.630 | 6.37 | | p = 0.780 |
|  | (22.7) |  | (22.9) | |  |
| **HH_Income** | 1,235.6 | p = 0.005 | 1,183 | | p = 0.007 |
|  | (440.4) |  | (440.4) | |  |
| **Read_Urdu** | 85.8 | p = 0.725 | 82.14 | | p = 0.735 |
|  | (243.3) |  | (242.7) | |  |
| **HH_Size** | 33.3 | p = 0.698 | 29.23 | | p = 0.733 |
|  | (0.698) |  | (85.8) | |  |
| **Formal_Save** | -1,471.9 | p = 0.043 | -1,582 | | p = 0.030 |
|  | (725.9) |  | (727.2) | |  |
| **Constant** | 8,913.6 | p = 0.000 | 9,189 | | p = 0.000 |
|  | (1283.5) |  | (1291) | |  |
| ***Savings account*** |  |  |  | |  |
| **R_speech_Subsidy_Help** | 1.866 | p = 0.000 | 1.867 | | p = 0.000 |
|  | (0.146) |  | (0.146) | |  |
| **R_speech_Subsidy** | 1.113 | p = 0.000 | 1.115 | | p = 0.000 |
|  | (0.142) |  | (0.142) | |  |
| **R_speech_Help** | 0.652 | p = 0.000 | 0.653 | | p = 0.000 |
|  | (0.146) |  | (0.146) | |  |
| **R_speech** | 0.419 | p = 0.005 | 0.422 | | p = 0.004 |
|  | (0.148) |  | (0.148) | |  |
| **C_speech_Subsidy_Help** | 0.633 | p = 0.000 | 0.633 | | p = 0.000 |
|  | (0.148) |  | (0.148) | |  |
| **C_speech_Subsidy** | 0.270 | p = 0.087 | 0.271 | | p = 0.087 |
|  | (0.158) |  | (0.158) | |  |
| **C_speech_Help** | 0.338 | p = 0.029 | 0.339 | | p = 0.029 |
|  | (0.155) |  | (0.155) | |  |
| **Age** | 0.010 | p = 0.004 | 0.010 | | p = 0.004 |
|  | (0.004) |  | (0.004) | |  |
| **HH_Income** | 0.0244 | p = 0.740 | 0.0244 | | p = 0.741 |
|  | (0.074) |  | (0.074) | |  |
| **Read_Urdu** | -0.019 | p = 0.629 | -0.019 | | p = 0.631 |
|  | (0.040) |  | (0.040) | |  |
| **HH_Size** | 0.012 | p = 0.387 | 0.012 | | p = 0.386 |
|  | (0.014) |  | (0.014) | |  |
| **Formal_Save** | -0.236 | p = 0.027 | -0.236 | | p = 0.027 |
|  | (0.107) |  | (0.107) | |  |
| **Constant** | -0.236 | p = 0.000 | -0.747 | | p = 0.000 |
|  | (0.206) |  | (0.206) | |  |
| $\boldsymbol{\rho}$ | 0.148 | p = 0.174 | 0.143 | | p = 0.195 |
|  | (0.109) |  | (0.109) | |  |
| **Observations** | 1,786 | | | 1,786 | |
| **Notes:** This table reports the output of the Heckman selection model using the ‘Total amount deposited in March to May’ as the outcome variable, instead of ‘Total savings at the end of May’ (i.e., the saving closing balance in May). R_speech = Religious speech; C_speech = Conventional speech; Subsidy = receipt of subsidy for opening the account; and Help = assistance with filling in application forms. In Column 2 the reference group is ‘No control’, meaning that the coefficients for Joint and Full control are relative to the group that has no control over the account. | | | | | |

**Section A8: The association between the received Mudarabah and level of Control**

**Table A8: Profits from the Mudarabah arrangement and Control – Heckman selection model**

| **VARIABLES** | **Heckman selection model** | | |
| --- | --- | --- | --- |
| ***Total received profit (March to May)*** | |  |  |
| **Control** | 11.00 | p = 0.054 |  |
|  | (5.71) |  |  |
| **Age** | 0.154 | p = 0.557 |  |
|  | (0.262) |  |  |
| **HH_Income** | 15.67 | p = 0.002 |  |
|  | (5.07) |  |  |
| **Read_Urdu** | 2.52 | p = 0.365 |  |
|  | (2.78) |  |  |
| **HH_Size** | 15.67 | p = 0.700 |  |
|  | (5.07) |  |  |
| **Formal_Save** | -11.04 | p = 0.188 |  |
|  | (8.39) |  |  |
| **Constant** | 74.7 | p = 0.000 |  |
|  | (14.36) |  |  |
| ***Savings account*** |  |  |  |
| **R_speech_subsidy_help** | 2.254 | p = 0.000 |  |
|  | (0.172) |  |  |
| **R_speech_Subsidy** | 1.479 | p = 0.000 |  |
|  | (0.170) |  |  |
| **R_speech_Help** | 0.962 | p = 0.000 |  |
|  | (0.175) |  |  |
| **R_speech** | 0.532 | p = 0.004 |  |
|  | (0.183) |  |  |
| **C_speech_Subsidy_Help** | 0.995 | p = 0.000 |  |
|  | (0.175) |  |  |
| **C_speech_Subsidy** | 0.657 | p = 0.000 |  |
|  | (0.182) |  |  |
| **C_speech_Help** | 0.694 | p = 0.000 |  |
|  | (0.181) |  |  |
| **Age** | 0.008 | p = 0.038 |  |
|  | (0.004) |  |  |
| **HH_Income** | 0.021 | p = 0.790 |  |
|  | (0.077) |  |  |
| **Read_Urdu** | -0.0039 | p = 0.926 |  |
|  | (0.041) |  |  |
| **HH_Size** | 0.007 | p = 0.604 |  |
|  | (0.014) |  |  |
| **Formal_Save** | -0.222 | p = 0.045 |  |
|  | (0.111) |  |  |
| **Constant** | -2.01 | p = 0.000 |  |
|  | (0.232) |  |  |
| $\boldsymbol{\rho}$ | 0.1086 | p = 0.301 |  |
|  | (0.1052) |  |  |
| **Observations** | 1,743 | | |
| **Notes:** This table reports the output of the Heckman selection model using the ‘Total amount received in Mudarabah (i.e., profits) from March to May’ as the outcome variable, instead of ‘Total savings at the end of May’ (i.e., the saving closing balance in May). R_speech = Religious speech; C_speech = Conventional speech; Subsidy = receipt of subsidy for opening the account; and Help = assistance with filling in application forms. | | |  |

**Section A9: Participants who are likely to be substituting savings from other sources for money in their Asaan Savings account**

**Table A9a: Change in total level of savings since opening the Asaan savings account**

|  | **No control** | | **Control** | |
| --- | --- | --- | --- | --- |
|  | **Savings in other sources not lower** | **Savings in other sources lower** | **Savings in other sources not lower** | **Savings in other sources lower** |
| **Same or lower**  **total savings** | 40 | 16 | 149 | 86 |
| **Higher total savings** | 27 | 10 | 85 | 45 |
| **Total** | 67 | 26 | 234 | 131 |
| **Missing values** | 66 | | 40 | |
| **Total in group** | 107 | | 405 | |
| **Notes:** The information presented in this table is based on the responses to the survey questions: “If you consider all your savings, informal as well as formal, is the total amount that you save per week/ month/year higher now that you have a bank account?” and “Are you saving less per week, month or year in other ways, formal and informal, now that you have a bank account?”, participants were asked in the follow-up survey. | | | | |

**Table A9b: Change in the level of savings per savings instrument since opening the Asaan**

**savings account**

|  | **No control** | **Control** | **No control** | **Control** |
| --- | --- | --- | --- | --- |
| **Money in mobile account** | 10 | 31 | 9% | 8% |
| **Prize bonds** | 5 | 18 | 5% | 4% |
| **Formal committee** | 0 | 1 | 0% | 0% |
| **Informal committee** | 0 | 12 | 0% | 3% |
| **Keep cash at home** | 3 | 31 | 3% | 8% |
| **Jewelry/gold** | 3 | 22 | 3% | 5% |
| **Invest in business** | 5 | 31 | 5% | 8% |
| **Lend to relatives** | 7 | 17 | 7% | 4% |
| **Total respondents** | 33 | 163 | 31% | 40% |
| **Total missing values** | 74 | 242 | 69% | 60% |
| **Total in group** | 107 | 405 | 100% | 100% |
| **Notes:** The information presented in this table is based on the responses to the survey question: **“**In which of the following forms of formal and informal saving instruments are you saving less/have less money invested now as a result of opening a bank account?”, participants were asked in the follow-up survey. | | | | |

**Section A10: Customer satisfaction**

**Table A10: Customer satisfaction with willingness of customer service representatives to listen and respond to their needs across control**

|  | **No control** | **Control** | **No control** | **Control** |
| --- | --- | --- | --- | --- |
| **Very satisfied** | 0 | 0 | 0% | 0% |
| **Satisfied** | 31 | 142 | 29% | 35% |
| **Neutral** | 49 | 153 | 46% | 38% |
| **Dissatisfied** | 27 | 110 | 25% | 27% |
| **Very dissatisfied** | 0 | 0 | 0% | 0% |
| **Total** | 107 | 405 | 100% | 100% |
| **Notes:** The information presented in this table is based on the responses to the survey question: **“**In terms of services you receive from customer service representatives, how satisfied are you with their willingness to listen and respond to your needs?”, participants were asked in the endline survey. | | | | |

1. There is only one missing value for the control variable ‘Formal_Save’, which captures whether the participant had formal savings prior to this study. [↑](#footnote-ref-1)
2. Since our sample includes only married female participants, it seems sufficiently safe to assume that there are no participants who jointly decide over the use of savings with household members other than their husband only. In other words, the category ‘no control’ most likely only incorporates participants who have no decision-making power over the use at all. [↑](#footnote-ref-2)
3. The inverse Mills ratio is defined as λ = ρ*σ, where ρ is the correlation between the error term of the selection specification and the error term of the second stage regression. σ provides information about the standard errors. In the two-step Heckit models, the Wald test of independent equations tests whether the inverse Mills ratio is equal to zero instead of whether ρ equals zero. [↑](#footnote-ref-3)
